# From Crosstalk between Immune and Bone Cells to Bone Erosion in Infection

**DOI:** 10.3390/ijms20205154

**Published:** 2019-10-17

**Authors:** Gaurav Kumar, Pierre-Marie Roger

**Affiliations:** 1 Unité 576, Institut National de la Santé et de la Recherche Médicale, 06200 Nice, France; roger@elsan.care; 2 Arthritis and Clinical Immunology, Oklahoma Medical Research Foundation, Oklahoma City, OK 73104, USA; 3 Service d’Infectiologie, Hôpital Archet 1, Centre Hospitalier Universitaire de Nice, Université de Nice Sophia-Antipolis, 06200 Nice, France

**Keywords:** bone erosion, bone infection, bone remodeling, osteoclasts, signaling crosstalk, T cells

## Abstract

Bone infection and inflammation leads to the infiltration of immune cells at the site of infection, where they modulate the differentiation and function of osteoclasts and osteoblasts by the secretion of various cytokines and signal mediators. In recent years, there has been a tremendous effort to understand the cells involved in these interactions and the complex pathways of signal transduction and their ultimate effect on bone metabolism. These crosstalk mechanisms between the bone and immune system finally emerged, forming a new field of research called osteoimmunology. Diseases falling into the category of osteoimmunology, such as osteoporosis, periodontitis, and bone infections are considered to have a significant implication in mortality and morbidity of patients, along with affecting their quality of life. There is a much-needed research focus in this new field, as the reported data on the immunomodulation of immune cells and their signaling pathways seems to have promising therapeutic benefits for patients.

## 1. Introduction

Inflammation due to bone infection is a complex cascade of events, being initiated on the entry of the pathogen inside the host to eliminate the invading pathogen and also to protect the host from tissue damage. The process occurs due to the well-coordinated activity of cells of the innate and adaptive immune system in crosstalk with multiple cytokines and chemokines. However, prolonged and uncontrolled immune activation under pathological conditions may lead to autoimmune diseases, leading to bone and other soft tissue damage [1,2].

Osteoimmunology, the terminology coined in 2000 by Aaron and Choi, is an emerging field of research focusing on the interaction between immune cells and the skeletal system [3]. After a decade of research, it seems that almost all immune cells are capable of communicating with bone cells and vice versa and the interdisciplinary approach may lead to the development of targeted therapies [1,3]. More particularly, there exists a close interaction and cross-talk mechanism between the bone forming cells (osteoblasts) the bone resorbing cells (osteoclasts) and the T cells of the adaptive immune system [4,5].

In this review, we will focus on the interactions and cross-talk between various cells of the innate and adaptive immune system, with osteoclasts, leading to bone erosion in inflammation due to infection.

## 2. Epidemiology

Bacterial diseases that affect the bones include osteomyelitis, periodontitis, periapical infection, septic arthritis, and others [1,6]. Osteomyelitis affects about 2 out of every 10,000 people, including both children and adults. Osteomyelitis leads to inflammatory bone loss and is a subject of recurrence as the infecting bacteria acquires the ability to evade host defenses and resist antimicrobial therapy, as seen mainly in the case of chronic *Staphylococcus* infections related to surgical prosthetic devices. This is considered as one of the main causes behind loosening of the implant [7,8]. Bone infection at poorly vascularized sites is often difficult to treat and requires a prolonged and intensive antimicrobial therapy along with surgical drainage or debridement. In the majority of bone and joint infections, gram-positive organisms, particularly, *Staphylococcus aureus*, are the main causative microbes [9,10]. *Staphylococcus aureus* is the major infecting microbe accounting for approximately 50% cases of human osteomyelitis because of its capacity to express bacterial adhesion molecules, that aid in attachment to extracellular bone matrix. Also, it possesses the ability to evade host defenses, attack host cells, and colonize bone persistently [11,12]. In immunosuppressed and sickle-cell patients, *Salmonella* species are the common causative agents leading to bone infection [13,14]. Gram-negative bacteria are rarely found in bone infections, but some specialized populations have been reported to cause septic arthritis, such as *Haemophilus influenzae* in children and *Neisseria gonorrhea* in young adults [10]. Bacterial infection of prosthetic implants is another serious bone complication for which the most common causative microbes are *Staphylococcus aureus* or *coagulase–negative staphylococci* [15]. Currently, it is estimated that up to 2.5% of primary hip and knee arthroplasties and up to 20% of revision arthroplasties are complicated by periprosthetic joint infection [16]. 

## 3. Osteoblasts and Osteoclasts

Osteoblasts are the specialized bone forming cells that originate from pluripotent mesenchymal stem cells and functions mainly to produce bone matrix proteins and mineralization of bones, apart from expressing osteoclastogenic factors. RUNX2 (runt-related transcription factor 2) is necessary for their development and differentiation, as RUNX2-deficient mice lack mineralized bone tissues due to a block in osteoblast maturation [17,18].

Osteoclasts are tissue-specific giant polykaryons derived from the monocyte/macrophage hematopoietic lineage and are the only cells capable of breaking down mineralized bone, dentine, and calcified cartilage [19,20]. The presence of receptor activator of NF-κB ligand (RANKL) and macrophage-colony-stimulating factor (M-CSF) are essential for the maturation and fusion of multinucleated cells leading to the formation of functional osteoclasts, that express osteoclast specific markers such as tartrate-resistant acid phosphatase (TRAP), cathepsin K, calcitonin receptor (CTR), and integrin receptors [21,22]. 

## 4. Bone Formation and Remodelling

Bone is a multifunctional organ acting as the center for hematopoiesis, apart from serving as the principal locomotory system and providing structural support for internal organs. It also acts as a reservoir of calcium and phosphorous necessary to maintain the body’s mineral homeostasis. Bone formation and skeletal growth is achieved by two main processes, commonly known as modelling (uncoupled) and remodelling (coupled). Modelling occurs as a process to maintain normal bone physiology and growth, where the osteoblasts form the bones and the osteoclasts resorb the bone matrix. These processes occur in an independent manner in different parts of the body i.e., bone formation is not dependent on bone resorption. However, bone remodelling involves a complex network of specialized cells forming the basic multicellular unit (BMU) which consists of osteoclasts, osteoblasts, mature osteoblasts (osteocytes), and the capillary blood supply [23,24]. 

The remodelling process occurs during infection, repair, and regeneration of bone in which the bone resorption and bone formation are coupled and tightly regulated. The initiation of this process starts with the recruitment of osteoclast precursor cells, which differentiate and mature into osteoclasts to maintain the bone resorption activity. A reversal process then occurs in which the bone resorbing osteoclast activity subsides and the osteoclasts secrete sphingosine 1–phosphate, which induces the recruitment of osteoblasts. The osteoblasts then come into action for bone formation and are further fully differentiated to become osteocytes [24]. These osteocytes remain embedded in the bone matrix and regulate the process of bone remodelling [25].

Children have high bone turnover rate where bone formation exceeds bone resorption, whereas in young adults, this turnover is approximately very well balanced. With ageing, this turnover gets reversed and bone resorption increases compared to bone formation, thus leading to a net bone loss. A defective remodelling process leads to various bone metabolic diseases such as osteoporosis, Paget’s disease of bone, osteopetrosis, and osteogenesis imperfect [20]. Also, the differentiation and activity of osteoblasts and osteoclasts are dependent on the body’s immune system. Therefore, a complex crosstalk and interaction between bone cells and immune cells takes place for a necessary and tightly regulated bone remodelling process. The osteoclasts are the specialized cells that solely carry out the function of bone resorption and our aim is to discuss the mechanism leading to bone infection and erosion. Therefore, in this review we will discuss the interaction of bone cells with immune cells which affects osteoclasts function during the bone resorption process.

## 5. Calcineurin/Nuclear Factor of Activated T Cells: An Important Signaling Pathway Associated with Osteoclastogenesis and Regulation of Immune Cells

The nuclear factor of activated T cells (NFAT) family is composed of five transcription factors that include NFATc1-4 and NFAT5. Calcineurin (CN), a key phosphatase, regulates cell proliferation, differentiation and development by aiding the translocation of NFAT into the nucleus, except NFAT5 [26]. The process of osteoclastogenesis is initiated by RANKL-mediated activation of the CN/NFAT signaling pathway. CN/NFAT pathway activation induces NFATc1 translocation from the cytoplasm to the nucleus, where it transcribes osteoclast specific genes such as TRAP, CTR and osteoclast-associated receptor leading to the differentiation and maturation of osteoclasts. The regulatory functions of NFATc1 in the osteoclastogenesis process were evident due to the fact that deletion of *NFATc1* in mice resulted in poor osteoclast development [27]. In a wear particle-induced inflammation model, inhibition of RANKL induced activation of CN/NFAT pathway also inhibited osteoclastogenesis and thereby protected the loosening of implants [28].

CN/NFAT signaling pathway, in addition to regulating osteoclastogenesis, also regulates the development and function of immune cells. This pathway is implicated in the activation of T cells through T cell receptor for the induction of antigenic and tolerogenic functions [29]. NFATc1 primarily stimulates T cells, whereas NFATc2 may have both stimulatory and inhibitory role in T cell activation and differentiation [30,31]. In addition to T cells, this pathway also regulates the development of early as well as mature B cells. Lack of NFATc1 in the early phase of B cell development results in severe B cell lymphopenia due to the inability of pro-B cells to transition into pre-B cells. In the mature B cell subsets, NFATc1 regulates the proliferation of splenic B cells and inhibits their regulatory functions by inhibiting IL-10 production [32]. NFAT signaling in dendritic cells (DCs) challenged with bacteria induces the production of IL-2, which further activates T cells and natural killer cells [33]. Defective calcineurin signaling in macrophages has been shown to exhibit lipopolysaccharide (LPS) tolerance, thereby suggesting a role for CN/NFAT pathway in mediating LPS tolerance [34]. Therefore, CN/NFAT pathway could be exploited for regulating osteoclastogenesis during bone infection.

## 6. Infection Initiates the Cross-Talk between Osteoclasts, Osteoblast, and Immune Cells

In *S. aureus* infected murine model, the cell surface-associated material commonly known as SAM, which mainly contains proteins, are capable of stimulating bone resorption, as evident by their ability to induce fibroblasts and monocytes to release osteolytic cytokines [35,36]. Bacteria also directly mediate bone destruction by inducing the apoptosis of osteoblasts. In a separate coculture model, *S. aureus* and *Salmonella* have been reported to induce TRAIL (Tumor Necrosis Factor-Related Apoptosis-inducing Ligand) in both mouse and human osteoblasts, thereby leading to their apoptosis [37].

Invading pathogens, specifically *Staphylococcus* and *Salmonella*, which to date remain the most studied bacteria in bone infection because of their prevalence in various human infectious diseases, have also adopted some indirect mechanism to activate the immune system leading to bone resorption. Bacteria have been reported to colonize osteoblasts, persistently live inside them, and activate them by secreting immune modulating proteins [38]. These activated osteoblasts secrete monocyte chemoattractant protein-1 (MCP-1) and T cell chemoattractant CXCL-10, to bring monocyte and T cells, respectively, at the site of infection. This was evident by cultured human and murine osteoblasts, which on exposure to *Staphylococcus* or *Salmonella* led to the secretion of these immune modulating factors [39]. MCP-1 activates T cells which then express RANKL and induce cell types such as fibroblast to express RANKL, finally leading to RANKL-mediated bone resorption [40,41].

**Figure 1:**
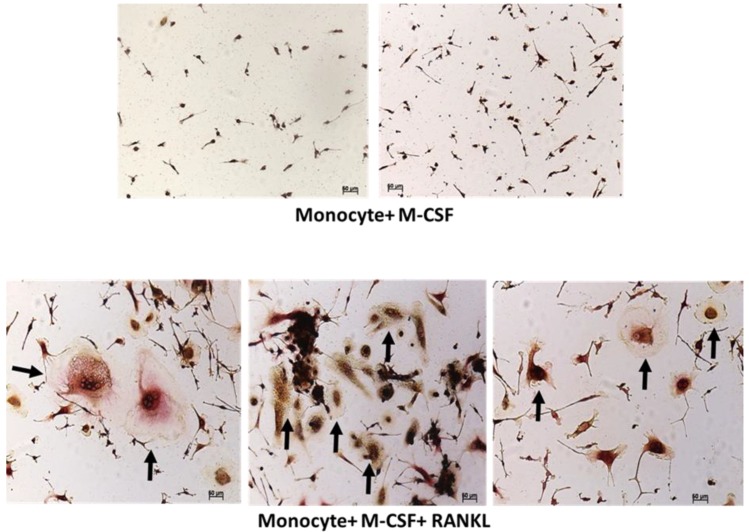
Differentiation of blood monocytes into osteoclast. Peripheral blood mononuclear cells (PBMCs) were isolated from blood of healthy donors by ficoll separation and monocytes were then purified from PBMCs. Purified monocytes were cultured for 15 days in the presence of macrophage-colony-stimulating factor (M-CSF) with or without receptor activator of NF-κB ligand (RANKL). Finally cells were stained for tartrate-resistant acid phosphatase (TRAP), an enzymatic marker for osteoclast identification. Cells were visualized using a Zeiss light microscope at 10 × resolution. Addition of RANKL to the culture medium led to the differentiation of monocytes into multinucleated large sized pink colored osteoclasts. Black arrows indicate osteoclasts.

**Figure 2:**
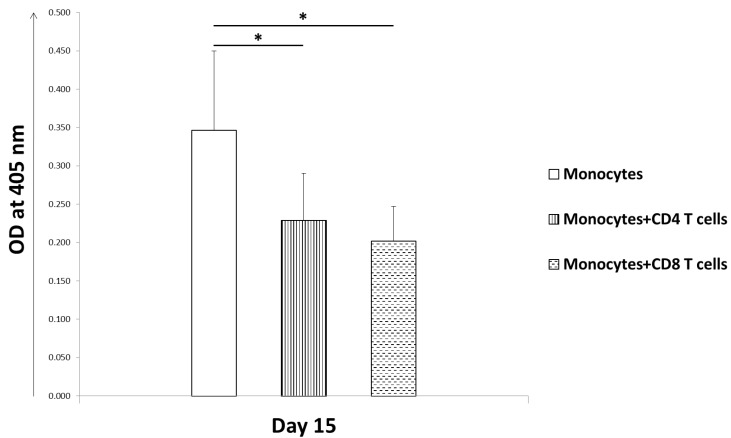
Human T cells inhibit osteoclast formation *in vitro*. Blood monocytes were differentiated into osteoclasts in the presence of macrophage-colony-stimulating factor (M-CSF) and receptor activator of NF-κB ligand (RANKL) for 15 days. T cells derived from human bone tissue, by cutting bones into fines pieces and separating cells by centrifugation, were separated into CD4 and CD8 T cells by flow cytometer. Addition of these isolated CD4 or CD8 T cells separately to the differentiating blood monocytes led to a decrease in the number of osteoclasts being formed in vitro cellular coculture model when observed after 15 days post culture. A comparative method of colorimetric assay was used to determine the number of osteoclasts formed by measuring the TRAP activity through absorbance, which is a prominent osteoclast enzyme. The data is from three independent experiments. Student *t* tests were used to determine statistical significance (* *p* < 0.05).

For the differentiation and function, osteoclasts requires mainly three factors, M-CSF, RANK, and its ligand RANKL which are actively produced by bone marrow stromal cells, osteoblasts, and T cells. Accordingly, we differentiated peripheral blood mononuclear cells (PBMC) derived monocytes into osteoclasts in the presence of M-CSF and RANKL for 15 days. Fully differentiated osteoclasts were formed after 15 days, which were morphologically large multinucleated cells and stained positive for TRAP (Figure 1). To further study the effect of CD4 and CD8 T cells on the formation of osteoclasts, we cocultured them together. When resting CD4 and CD8 T cells from healthy PBMC were purified and cocultured with differentiating monocytes, we observed a reduction in the number of osteoclasts being formed. PBMC-derived CD4 and CD8 T cells from bacterial bone infected patients also showed similar inhibition of osteoclast formation in cellular coculture model. Interestingly, CD8 T cells showed greater inhibition of osteoclastogenesis as compared to CD4 T cells (Figure 2).

M-CSF, a secreted or transmembrane cytokine produced by osteoblasts, binds to c-Fms expressed by pre-osteoclast cells and induces their proliferation and differentiation into fully functional osteoclasts [42,43]. M-CSF binding with c-Fms further induces downstream transcription factors c-Fos and PU.1. c-Fos is a component of a transcription factor complex which is necessary for the differentiation and proliferation of osteoclast and macrophages [44], whereas PU.1 is a transcription factor regulating proteins which are essential for the development of myeloid cells and their osteoclast phenotype [45]. M-CSF deficient mice showed absence of macrophages and osteoclasts, whereas c-Fos and PU.1 deficient mice had inhibition of osteoclastogenesis and hence osteopetrotic phenotype. Osteopetrosis is a bone disease characterized by skeletal fragility despite increased bone mass. 

RANKL is another prominent factor that is expressed and produced as a soluble cytokine by osteoblasts, osteocytes, T cells, and endothelial cells. RANKL binds to RANK expressed on the surface of pre-osteoclast cells, thereby initiating a signaling cascade leading to their differentiation into functional osteoclasts via the Nuclear factor-kappa B (NFκB) mediated pathway [46]. Severe osteopetrosis was observed in both RANK and RANKL deficient mice due to the depletion of osteoclasts [47,48]. Nadia et al. reported that RANKL administration to RANKL^−/−^ mice, which have an osteopetrotic phenotype and lack osteoclasts, can restore bone resorption and ameliorate skeletal defects [49]. 

**Figure 3:**
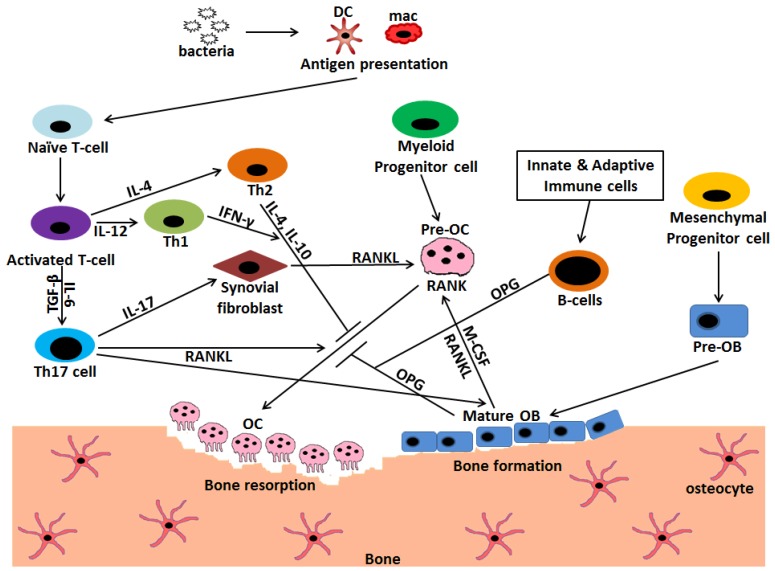
Interaction and crosstalk between immune cells, osteoblasts, and osteoclasts mediated via different cytokines regulate the extent of bone erosion during infection. Osteoclasts (OC) are the cells of myeloid origin that degrade the bone matrix, whereas osteoblasts (OB) are the bone forming cells that have mesenchymal origin. Bacterial entry into the host initiates a complex crosstalk between immune cells, mainly, T and B cells with osteoclasts. The invading pathogen is phagocytized and presented by macrophages (mac) and dendritic cells (DC) to activate T cells. These activated T cells further get differentiated into T helper (Th) 1, Th2, and Th17 subsets. Th17 is the prominent osteoclastogenic T cell subset which expresses receptor activator of NF-κB ligand (RANKL) and induces the formation of osteoclasts by binding to RANK on pre-osteoclasts. It also secretes IL-17 that induces the synovial fibroblasts as well as osteoblasts to express RANKL further leading to osteoclastogenesis. Contrarily, Th1 and Th2 subset of T cells inhibits osteoclastogenesis by secreting cytokines INF-γ, IL-4 and IL-10 respectively. B cells, being activated by innate and adaptive immune cells, secrete OPG, which acts as an inhibitor to osteoclast formation process. Osteoblasts also secrete macrophage-colony-stimulating factor (M-CSF) and RANKL that aids in the process of osteoclastogenesis. 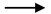 Stimulation; 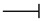 Inhibition.

Osteoblasts also produce osteoprotegrin (OPG), which acts as a decoy receptor and binds RANKL, which in turn inhibits RANK–RANKL association and thereby osteoclast formation [50]. OPG deficient mice (OPG^−/−^) and RANKL overexpressing transgenic mice (RANKL-Tg) both exhibited an osteoporosis phenotype due to enhanced osteoclastogenesis, but OPG^−/−^ mice have enhanced bone resorption as compared to RANKL–Tg due to a higher RANKL/OPG ratio in OPG^−/−^ mice [51,52]. Masanori et al. showed that the addition of anti-RANKL, such as bisphosphonates, significantly prevented alveolar bone loss in OPG^−/−^ mice and administration of OPG to RANKL-Tg mice also showed a similar effect [53]. Therefore, RANKL signal transmission fully depends on the ratio of RANKL/OPG and RANK expression on osteoclast precursor cells. After differentiation and maturation, osteoblasts may differentiate into osteocytes, undergo apoptosis, or become cells covering the bone surface, thereby protecting the bone matrix from coming into direct contact with osteoclasts to prevent bone erosion [54] (Figure 3). 

## 7. Biofilm and Host Cell Interaction Leads to Loosening of Implants

Infection associated with orthopedic implants remains the most severe risk for bone infection. Orthopedic implants act as a site for bacterial colonization and wear particles from these implants cause infection related inflammation. In the majority of implant-associated infections, *S. aureus* is the main causative pathogen, due to its ability to adhere to the implant surface, grow, and form biofilms [55]. These biofilms protect the residing bacteria from phagocytosis or killing by immune cells because of the inability of immune cells to penetrate them. On the other hand, bacterial factors such as pathogen-associated molecular patterns, interact with immune cells and induce the release of inflammatory cytokines such as TNF, IL-1, and IL-6. As a consequence, RANKL expression is increased by osteoblasts, leading to RANKL-mediated increased bone resorption [55,56]. 

Neutrophils are the first line of defense against bacterial biofilm, but they are unable to effectively clear the biofilm bacteria. Biofilm also has the ability to skew the infiltrating macrophages from M1 (pro-inflammatory) to M2 (anti-inflammatory) subtypes. This skewing of macrophages further prevents the killing of embedded bacteria [57]. Studies using human samples reported the presence of T cells, mostly the activated cytotoxic T cells, at the site of biofilm formation. However, decreased T cell proliferation at the biofilm suggests that only a few T cells are able to mount a strong immune attack for clearing bacteria [58,59]. Together, these results suggest that biofilms could induce osteoclastogenesis as well as evade the host immune attack, thereby leading to implant associated bone erosion. 

## 8. Mimicking Function, a Strong Support Favoring Osteoimmunology

Recent research work led to interesting findings that osteoclasts in both human and mice have the ability to mimic some of the properties of T regulatory cells (Tregs). Li et al. reported that human osteoclasts express MHC class I and II along with costimulatory markers and have the property to induce antigen-specific CD4 and CD8 T cell responses [60].Mouse osteoclasts have been reported to express Class I MHC molecules and mimic CD8 T cell activation. These activated CD8 T cells express forkhead box P3 (Foxp3), a marker specific for Tregs and which has the potential to suppress antigen specific T cell proliferation [61]. Further, these specific CD8 T cells mimicking Tregs were shown to inhibit osteoclast formation via secretion of cytokines mainly INF-γ, IL-6, and IL-10 in a similar way to normal activated T cells seen in arthritis model [62]. Axmann et al. reported that Tregs are able to inhibit osteoclast differentiation via cytotoxic T-lymphocyte-associated protein-4 (CTLA-4) mediated pathway [63]. Together, these findings indicate that bone physiology and homeostasis is the net effect of close interactions between bone cells and immune cells, during which both exploit and utilize each other’s signaling pathways for their own purpose and function. 

## 9. Innate and Adaptive Immune Cells Modulate Bone Resorption during Infection

Coordinated activation of the innate and adaptive immune system is essential for the eradication of invading bacteria during bone infection. During this inflammatory reaction, various cytokines are also released by these cells, which in turn affect the bone remodelling process apart from initiating a protective host immune response. In this section, we will discuss in detail the immune cells, particularly T cells and their signaling mediators that affect osteoclastogenesis during bone infection.

### 9.1. T Cells

T cells are the major cells of the adaptive immune system and are crucial mediators of the immune response. T cells originate and differentiate mainly into CD4 and CD8 T cells in thymus from lymphoid progenitor cells, which develop from hematopoietic stem cells in the bone marrow [64,65]. A small proportion of T cells get differentiated into natural killer T cells (NKT) which function by eliciting immune responses mainly to pathogens, but are also implicated in autoimmunity and graft rejection [66].

Activated T cells express RANKL, which directly affects osteoclast precursor cells and induces the formation of osteoclast or osteoclastogenesis *in vitro*. In contrast, resting T cells have been reported to play a protective role in bone resorption. T cell deficient mice had no effect on RANKL mRNA expression, but the mice showed increased osteoclast numbers and reduced bone density [66]. In an in vitro coculture of murine bone marrow cells, John et al. reported that though CD4 T cells had no effect on osteoclastogenesis, depletion of CD8 T cells led to a 40% increase in osteoclast formation [67].

The inhibitory effect of resting T cells on osteoclast formation seems to be mediated through involvement of B cells, as depletion of CD4 and CD8 T cells in mice led to increased osteoclastogenesis by a mechanism that involved the complete suppression of OPG production by B cells [68]. Also, B cell deficient mice showed increased osteoclast formation and bone resorption, as these cells are the main OPG producing cells [66].

T cell infiltration has been implicated in various bone infection diseases as well as in autoimmune diseases, such as periodontitis and rheumatoid arthritis (RA). As bone loss occurs in these diseases and osteoclast-like cells were reported to be present at the site of infection, these data were strongly correlated to the role of osteoclasts in bone resorption [69,70]. Infection-induced activation of T cells leading to increased RANKL expression further contributed to increased osteoclast formation and bone resorption [71].

Cytokines produced by T cells also play a prominent role in bone physiology and metabolism. INF-γ, a major cytokine produced by T helper1 (Th1) cells, inhibits osteoclast formation and bone erosion along with IL-12 and IL-18, which induces Th1 cell differentiation [72]. This was evident, as mice deficient in INF-γ receptor showed severe bone resorption in collagen induced arthritis [73,74]. Th2 cytokines, mainly IL-4 and IL-10 have also been reported to have an inhibitory effect on osteoclast formation [74,75] (Table 1).

#### 9.1.1. Tregs

Tregs are a specialized subset of CD4 T cells which are characterized by the expression of CD25 and Foxp3 and exhibit anti-inflammatory effects through the suppression of CD4 T effector cells. In a mouse model of implant infection, toxins produced by *S. aureus* led to early reduction in the frequency of Tregs. This early downregulation of Tregs increased the proinflammatory Th1 and Th17 immune response by secretion of IL-6 and IL-17 and inhibited Th2 response, thereby leading to chronic immune activation during bone infection [76]. Tregs also act as immunosuppressive and anti-inflammatory cells by secreting IL-10 and transforming growth factor-beta (TGF-β) [77]. CD28 expressed by T cells binds to CD80/CD86 for effective stimulation and activation of T cells, whereas CTLA-4, which also competes for the same ligand, leads to T cell suppression. Tregs also express CTLA-4, which binds to CD80/CD86 and inhibits T cell activation, and therefore T cell induced RANKL expression, and results in the inhibition of osteoclast formation [78]. These inhibitory effects were further verified by a coculture assay in which monocyte-differentiated osteoclasts showed less bone resorption in the presence of Tregs [79].

#### 9.1.2. Th17 Cells

Th17 cells are IL-17 producing helper T cells that are differentiated from naïve CD4 T cells, which protect the host from bacterial and fungal infections, in addition to playing a prominent role in autoimmune diseases through the secretion of inflammatory cytokines [5]. In mouse, Th17 cells are differentiated from naïve T cells with TGF-β and IL-6, whereas in humans, TGF-β, along with other inflammatory stimuli, such as IL-23, IL-6, and IL-1β, acts as the inducing factors. They produce a wide array of Th17 signature cytokines that includes IL-17A, IL-17F, IL-22 and IL-26, along with a subset producing a small amount of IFN-γ [80]. IL-17 expression has been reported to increase in RA joints and Th17 cells isolated from RA joints, expressed RANKL, and possessed the capacity to induce TNF-α production and RANKL expression on synovial fibroblasts. Sato et al. have shown that Th17 cells are the exclusive osteoclastogenic T cell subset, due to their capacity to express RANKL and to induce RANKL expression on mesenchymal cells, whereas Th1 and Th2 T cell subsets have marked anti-osteoclastogenic effects. Also, mice deficient in IL-17 or IL-23 showed no osteoclast mediated bone loss when challenged with LPS [75]. Thus, it seems that Th17 cells directly affect osteoclast formation by expressing RANKL and indirectly Th17 mediates inflammation and activation of immune cells by IL-17 secretion. Activation of immune cells leads to secretion of inflammatory cytokines mainly TNF-α and IL-1, which in turn induces RANKL expression on cells supporting osteoclastogenesis [81].

#### 9.1.3. T Cells Alter Costimulatory Molecules and Play a Protective Role in Bone Infection in Humans

**Figure 4:**
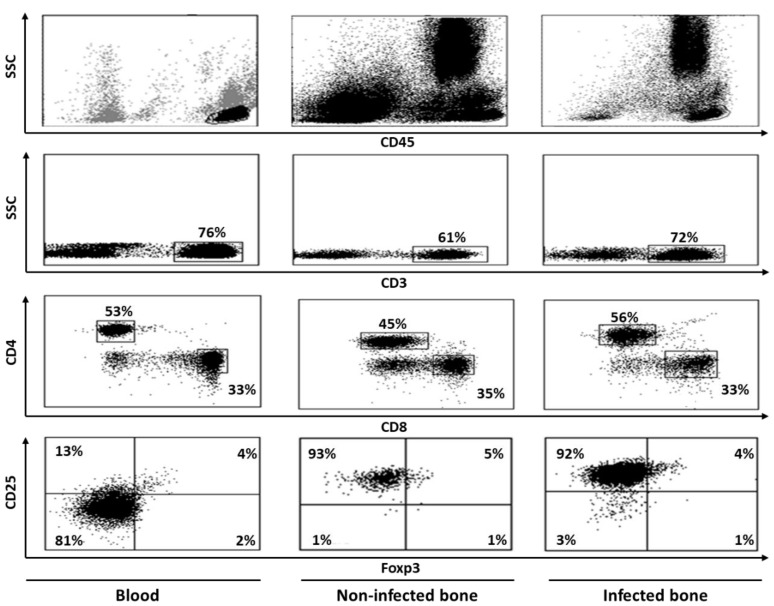
Flow cytometric dot plot images showing staining patterns of T cell populations in human blood, non-infected bone and infected bone. Peripheral blood mononuclear cells (PBMCs) were separated from blood by ficoll and bone cells were isolated by cutting bone samples into fine pieces, vortexing, filtering, and centrifugation. Cells were then labeled with various monoclonal antibodies, both extracellular as well as intracellular, and analyzed by flow cytometer and doing sequential analysis. Cells were first gated on CD45 and then CD3+ population were gated on these CD45+ cells. Finally, CD4+ and CD8+ T cell populations were identified by gating on CD3+ cells. Expressions of other markers were studied on these CD4 and CD8 T cell populations. CD4+ cells that were positive for CD25 and Foxp3 double markers were considered to be Tregs. Bone tissues that were without any infection were considered non-infected, whereas those with reported bacterial infection were considered to be infected.

In an attempt to characterize T cells at the site of human bone infection, we extensively studied cells in the bacterial infected cortical bones of the hip, present at the interface of bone marrow. Elevated T cell activation, along with reduced proliferation, was observed regardless of the T cell subsets, i.e., CD4 and CD8 T cells. Interestingly, we did not find significant levels of apoptosis and the presence of Tregs (Figure 4). A marked alteration of costimulatory molecule expression was observed. CD4 T cells showed reduced expression of CD28 during infection, whereas the expression of CTLA-4 remained unchanged. These CD28 negative CD4 T cells showed higher expression of perforin in comparison to their CD28 positive counterparts, indicating their cytotoxic potential. As reported earlier, we also observed an up-regulation of the CD40-CD40L pathway for both T cell subsets, which seems to be necessary for long-lasting activation of T cells, leading to bone resorption [58].

### 9.2. B Cells

B cells are well known to act as antigen presenting cells (APCs) and differentiate into antibody secreting plasma cells upon encountering pathogens [82,83]. As the maturation and differentiation of B cells takes place in the bone marrow in close proximity to bone cells, a complex interaction and cross-talk mechanism exists between the two, which affects their activity and function. Cytokines affecting bone metabolism, like TNF-α, IL-1 and IL-13, and vascular cell adhesion molecules, like molecules that are secreted by bone marrow stromal cells, directly affect B cell homing and differentiation [84]. Mice deficient in RANK or RANKL, the two major mediators of osteoclastogenesis, showed severe osteopetrosis along with reduced numbers of mature differentiated B cells secreting IgM and IgD in the lymph nodes and spleens, which could be related to reduced bone marrow cavities or altered stromal cells [50]. Immunomodulatory experimentation in mouse models altering RANKL/RANK/OPG pathways and interaction led to severe defects in B cell maturation and functions [85]. B cells expressed RANKL and differentiated into osteoclasts in the presence of M-CSF and RANKL during in vitro coculture [86]. B cell depletion inhibited inflammatory bone loss in patients with RA. [87]. *P. gingivalis* infection in mice resulted in significant increase of B cell numbers as well as RANKL expression on B cells. Interestingly, this was not observed in B cell deficient *μ*MT mice, which were protected from infection-induced bone resorption [88]. In another report, *P. gingivalis* induced experimental periodontitis, adoptive transfer of regulatory B cells significantly inhibiting periodontal bone resorption. The inhibitory effects on bone loss by adoptive transfer were associated with reduced production of RANKL/OPG, TNF-α, and IL-1β, and increased IL-10 secretion [89]. A detailed analysis of circulating B cell subsets in severe periodontitis showed an increase of memory B cells, mainly class switched memory B cells. In addition, RANKL expression on B cells were increased, but the number of B cells with regulatory functions were decreased in severe periodontitis [90]. Altogether, these evidence suggests an important regulatory role of B cells in bone erosion, and therefore, B cells could be a potential therapeutic target for infection-induced bone loss.

### 9.3. Neutrophils

Neutrophils are short-lived local inflammatory cells of the immune system that infiltrate bone inflammation sites in large numbers. Neutropenic patients lacking functional neutrophils are associated with early-onset periodontitis, thereby indicating that neutrophils are the first line of defense against microbes in bone infection [91,92]. Neutrophils have been reported to play an active role in the inflammatory process by secreting proteins and lipids in patients with RA and periodontitis, as well as in LPS-induced arthritis mouse models [93]. Drugs with therapeutic potential in RA patients, such as leflunomide and methotrexate, cause a reduction in neutrophil activity [94]. Human and murine neutrophils increase the surface expression of membrane bound RANKL through toll-like receptor (TLR) interaction after LPS stimulation, leading to enhanced osteoclatogenesis and bone resorption [95]. Also, the presence of neutrophils in the histology preparation of synovial tissues from RA patients indicate a possible cross-talk between neutrophils and osteoclasts [96,97]. In humans, depletion of neutrophils helps the bone repair process by increasing the expression of osteogenic factors at the site of bone injury. By living in close proximity to bone marrow stromal cells, neutrophils inhibits the synthesis of mineralized extracellular matrix. This may impair the bone healing process during inflammation in which there is increased influx of neutrophils [98]. Thus, neutrophils, not only act as proinflammatory cells during bone tissue inflammation, but also directly affect the bone erosion via osteoclast formation and activity.

### 9.4. Dendritic Cells

Dendritic cells are the most potent APCs and play a crucial role in the initiation and orchestering of adaptive immunity by selectively activating T cells and B cells in the lymphoid tissues. Osteoclasts and DCs share similarities between them as they both originate from a common monocyte/macrophage lineage. Though under steady state they are rarely localized in bone tissues and have no role in bone remodelling process, DCs can be transdifferentiated in the presence of MCS-F and RANKL into osteoclast *in vitro*, suggesting a direct involvement of DCs in osteoclastogenesis [99]. Bone loss in inflammatory condition has been reported by Alnaeeli et al. even though DC-deficient mice show no skeletal defects [100]. Under various pathological conditions such as RA and periodontal disease, DCs are reported to infiltrate at the site of infection and lead to T cell activation, or DCs can themselves differentiate into osteoclasts, thus leading to increased bone resorption [101]. Murine CD11c+ DCs can differentiate into osteoclasts that have the potential to induce bone resorption, both in vitro and in vivo [102]. Arizon et al. reported a protective immunoregulatory role for DCs in inflammation-induced bone resorption, in contrast to the earlier reports showing their osteoclastogenesis promoting functions. In *P. gingivalis* infected mice, loss of Langerhans cells (a subset of DCs) led to reduced Treg numbers, increased IFN-γ production and increased numbers of T cells expressing RANKL, which together resulted in enhanced bone resorption [103]. These data suggest that in inflammatory bone disorders, DCs not only act as an APCs to induce inflammation, but they could also function as osteoclast precursor cells that could contribute to enhanced bone resorption.

### 9.5. Macrophages

Macrophages are key cells of the innate immune system which, when activated by APCs, infiltrate inflammatory sites and phagocytize invading pathogens, further contributing to the process of eliminating microbes from the host body. Like DCs, macrophages also possess the capacity to differentiate into osteoclasts in the presence of MCS-F and RANKL when cultured *in vitro*. When activated in the synovium of joints, they actively produce pro-inflammatory cytokines such as TNF-α, IL-1 and IL-6, which directly contributes to the differentiation of osteoclasts and also acts to activate T cells by inducing RANKL expression on synovial fibroblasts. Macrophages also play a critical role in peri-implant osteolysis. Upon activation by wear particles, macrophages produce inflammatory cytokines IL-1, IL-6, TNF-α, and osteopontin. These inflammatory cytokines contribute to RANKL-induced osteoclastogenesis and bone loss [104]. In wear particle induced inflammation, M1 macrophages mainly produce IFN-γ, whereas M2 macrophages ameliorate debris-induced osteolysis [105].

## 10. Treatment Approach for Bone Infection Is Limited

Surgical debridement of the infected bone is generally performed for the management of osteomyelitis, followed by appropriate antimicrobial therapy for eradication of infection. Surgery is usually performed upon antibiotic failure, or in the case of chronic osteomyelitis, with necrotic bone and soft tissues [106]. Despite the use of antibiotic therapy and surgical debridement, the recurrence rate of chronic osteomyelitis is very high, sometimes even in 50 percent of cases [107]. Inflammation, cytokine secretion, and activation of immune cells, mainly T cells, are associated with bone resorption. Use of anti-inflammatory agents which block TNF or IL-6R to dampen the inflammatory cascade has resulted in limited repair of bone erosion [108,109]. A combination therapy including anti-TNF and OPG or parathyroid hormone has shown therapeutic potential in TNF-mediated arthritis in mice. The combination therapy completely reversed bone loss mainly by blocking osteoclasts and stimulating osteoblast function [110]. Alternatively, for a long-lasting and effective treatment approach, immune cells, and particularly T cells, should be targeted to exploit therapeutic potential, as they play a diverse role in the inflammatory process and bone erosion.

## 11. Conclusions

T cells, specifically Th17 cells, seem to occupy a central role in the interaction pathways of osteoblast formation, osteoclastogenesis, and bone remodelling. Many diseases of the bone have been linked to a component of immune cells with active participation. Since the role of T cells and its associated cytokines, such as TNF-α, IL-6 and IL-17 affect the bone remodelling process by modulating RANKL-RANK-OPG interaction, elucidating hierarchy of the regulatory network of cytokines, immune cells, and bone cells will be a promising approach towards elucidating treatments for bone defects.

The rapidly growing research work in the field of osteoimmunology is expected to provide a complete outline of the complex cross talk and interaction between different cells of the immune system with that of bone. A detailed knowledge of the involvement of the immune system, and the cytokines and signals mediated by them to control infection and bone resorption, will contribute significantly to drug development. Immunomodulating complex signaling pathways could be one aspect of cellular therapies which seems to have a high potential in the treatment of bone and joint diseases. With the recent advancements in deciphering the components involved in osteoimmunology, a great success has been achieved towards treating bone loss due to infection.

## Figures and Tables

**Table 1:** Major cytokines and osteoclastogenic mediators secreted by T cells and B cells that mediate bone resorption during infection. (RANKL = receptor activator of NF-κB ligand, OPG = osteoprotegerin, M-CSF = macrophage-colony-stimulating factor).

Cells	Cytokines and Mediators	Effect on Osteoclastogenesis
Th1	INF-γ	Inhibits
	TNF-α	Supports
Th2	IL-4	Inhibits
Th17	RANKL	Supports
	IL-17	Supports
Tregs	IL-10	Inhibits
	CTLA-4	Inhibits
B cells	OPG	Inhibits
Osteoblasts	RANKL	Supports
	M-CSF	Supports
	OPG	Inhibits
